# Sexual and Reproductive Health for Young Adults in Colombia: Teleconsultation Using Mobile Devices

**DOI:** 10.2196/mhealth.2904

**Published:** 2014-09-25

**Authors:** Catalina Lopez, Daniel Camilo Ramirez, Jose Ignacio Valenzuela, Arturo Arguello, Juan Pablo Saenz, Stephanie Trujillo, Dario Ernesto Correal, Roosevelt Fajardo, Cristina Dominguez

**Affiliations:** ^1^Center for Innovation and Health EducationFundacion Santa Fe de BogotaBogotaColombia; ^2^School of Medicine and Health SciencesUniversidad del RosarioBogotaColombia; ^3^Systems and Computing Engineering DepartmentUniversidad de Los AndesBogotaColombia; ^4^School of MedicineUniversidad de Los AndesBogotaColombia

**Keywords:** mobile health, youth and adolescents, telemedicine, remote consultation, Colombia, Latin America.

## Abstract

**Background:**

Sexual risk behaviors associated with poor information on sexuality have contributed to major public health problems in the area of sexual and reproductive health in teenagers and young adults in Colombia.

**Objective:**

To report our experience with the use of DoctorChat Mobile to provide sexual education and information among university students in Bogota, Colombia, and knowledge about the sexual risk factors detected among them.

**Methods:**

A mobile app that allows patients to ask about sexual and reproductive health issues was developed. Sexual and reproductive risk behaviors in a sample of young adults were measured before and after the use of the app through the validated survey Family Health International (FHI) Behavioral Surveillance Survey (BSS) for Use With Adults Between 15 and 49 Years. A nonprobabilistic convenience recruitment was undertaken through the study´s webpage. After completing the first survey, participants were allowed to download and use the app for a 6-month period (intervention), followed by completion of the same survey once again. For the inferential analysis, data was divided into 3 groups (dichotomous data, discrete quantitative data, and ordinal data) to compare the results of the questions between the first and the second survey. The study was carried out with a sample of university students between 18 and 29 years with access to mobile phones. Participation in the study was voluntary and anonymous.

**Results:**

A total of 257 subjects met the selection criteria. The preintervention survey was answered by 232 subjects, and 127 of them fully answered the postintervention survey. In total, 54.3% (69/127) of the subjects completed the survey but did not use the app, leaving an effective population of 58 subjects for analysis. Of these subjects, 53% (31/58) were women and 47% (27/58) were men. The mean age was 21 years, ranging between 18 and 29 years. The differences between the answers from both surveys were not statistically significant. The main sexual risk behaviors identified in the population were homosexual intercourse, nonuse of condoms, sexual intercourse with nonregular and commercial partners, the use of psychoactive substances, and lack of knowledge on symptoms of sexually transmitted diseases and HIV transmission.

**Conclusions:**

Although there were no differences between the pre- and postintervention results, the study revealed different risk behaviors among the participating subjects. These findings highlight the importance of promoting high-impact educational strategies on this matter and the importance of providing teenagers and young adults with easily accessible tools with reliable health information, regardless of their socioeconomic status.

## Introduction

Sexual risk behaviors associated with poor information on sexuality, such as the early onset of sexual intercourse and a high number of sexual partners, is one of the factors contributing to the status of sexual and reproductive health in teenagers and young adults as a public health problem [1,2].

In Colombia, the pregnancy rate was estimated to be 2.4 children per woman from 2002-2005, which is equivalent to 20 births per 1000 women. In teenagers, the pregnancy rate was estimated at 90 births per 1000 women (79 per 1000 in urban communities and 128 per 1000 in rural populations) [3]. This rate is one of the highest compared with the rest of Latin America and the United States. Regarding socioeconomic status (SES) as an influence factor for sexual risk behaviors, one study conducted in two cities in Colombia with 1100 teenagers from all socioeconomic backgrounds showed that there are indeed significant differences in patterns of sexual activity, cohabitation, and pregnancy between teenagers with varying SES in the country. The study showed a higher frequency of teenage pregnancy among low-level SES women, due to earlier onset of sexual intercourse, cohabitation, and less willingness to use contraceptive methods [4].

According to the National Demographic and Health Survey (ENDS) conducted by Profamilia in 2010 among 49,562 women between 15 and 49 years, it was determined that 99% had heard about HIV/AIDS, but knowledge was lower in those between 15 and 24 years old [5]. The level of knowledge was lower in rural areas (96%) than in urban areas (99%). Likewise, knowledge was lower among women who have no education (84%) and those at a lower economic level (95%). However, another study conducted in the country concluded that higher SES women share HIV and sexually transmitted diseases (STDs) risk characteristics with lower SES women, especially regarding cultural aspects and gender roles in relationships [6].

Various strategies of education for prevention have been explored, but their impact has not been as great as expected. From 2007, the Colombian Ministry of Health and Social Protection has been implementing the national adoption of the World Health Organization (WHO) Adolescent Friendly Health Services (AFHS) model. The model aims to facilitate the access and essential attention of young people and teenagers to sexual and reproductive health, in the context of the rights of health [7]. On the other hand, the United Nations Fund for Population Activities (UNFPA) Colombia promotes favorable conditions for the informed and protected exercise of sexuality in several cities of the country (not including Bogota). The UNFPA aims to do this by the promotion of comprehensive sexual and reproductive health services. The UNFPA also promotes the improvement of the socioeconomic determinants that contribute to HIV vulnerability [8,9]. Nonetheless, the rates of pregnancy and sexually transmitted diseases among young adults and teenagers have not decreased in the last 20 years in Colombia [8,10].

This situation has motivated the search for innovative programs through the use of emerging information technologies. These programs promise benefits in the diffusion of information and guidance toward prevention. However, their potential and impact have not been studied sufficiently in Latin America.

Specifically, in the field of sexual health for behavioral change, literature on the applicability of mobile technologies is limited. In 2010, The Cochrane Collaboration published their review on interactive, computer-based interventions for sexual health promotion [11]. In this report that analyzed 15 studies, a moderate positive effect was found on sexual health knowledge, a small effect on self-efficacy, and a small effect on sexual behavior. In the past years, there have been several studies and reviews that indicate that short message service (SMS) may be an effective low-cost method to promote sexual health among young people [12,13]. However, very few of these studies have been conducted in developing countries and most of them leave aside the use of mobile phone technologies and Web-based mobile apps. The WHO’s report “mHealth—New horizons for health through mobile technologies” [14] summarizes the global efforts through mobile devices for health promotion. The report showed many successful programs in health promotion using SMS even in Colombia, but it does not mention interactive mobile systems or mobile app-based programs. Yet it showed how this kind of system can raise awareness in sexual health problems and solutions. In 2006, Zhao et al concluded, “providing sexual education to students in Shanghai over the Internet is feasible and effective” [15]. His statements suggested that Web-based sexual education programs increased the students’ knowledge of reproductive health and led to significant changes in their attitudes toward sexuality, particularly on issues related to sexual freedom. The author concluded that the Internet offers significant potential to provide sexual education to students and teenagers in China. Furthermore, one study assessed the perception of a group of teenagers regarding technology for enhancing sexual health education, showing that young people can be enthusiastic and open to innovative ways of education [16].

Fundacion Santa Fe de Bogota is a private, nonprofit health organization located in Bogota, Colombia. The research group that conducted this study is part of the Education and Knowledge Management Department of the institution. This department is in charge of the Telehealth Center and all its related activities [17]. In September 2006, our research group started a Web-based medical counseling program service called DoctorChat. It is a free-access, online consulting service for Spanish-speaking users that allows them to submit health-related inquiries and to receive personalized and accurate responses from a well-known group of physicians through a simple, Internet-based form [18]. As previously reported [19,20], most queries were related to sexual health in the young population. Teleconsultation in Colombia has proved to be an innovative, low-cost, effective method to provide accurate and useful health information. The program also allows unrestricted open discussion on sensitive topics such as sexually transmitted diseases and sexual risk behaviors. With these findings we concluded that the expansion of the service into new platforms could enhance DoctorChat potential and positively impact key health indicators in Colombia, such as the rate of teenage pregnancy and spreading of diseases, through innovative educational services. With that in mind, the Web-based, mobile teleconsultation platform for DoctorChat was designed and developed in partnership with a software development group from the Universidad de los Andes, a private university in Colombia [21]. The ultimate goal of the app is to promote and deliver accurate sexual health information among young adults. Assessing a target population of young adults in Colombia, including their behaviors and knowledge regarding sexual health, can lead to future innovative interventions on sexual health education, thereby increasing its success. In this study, we tested the intervention in this convenience sample before expanding access to the intervention by a broader population.

**Figure 1:**
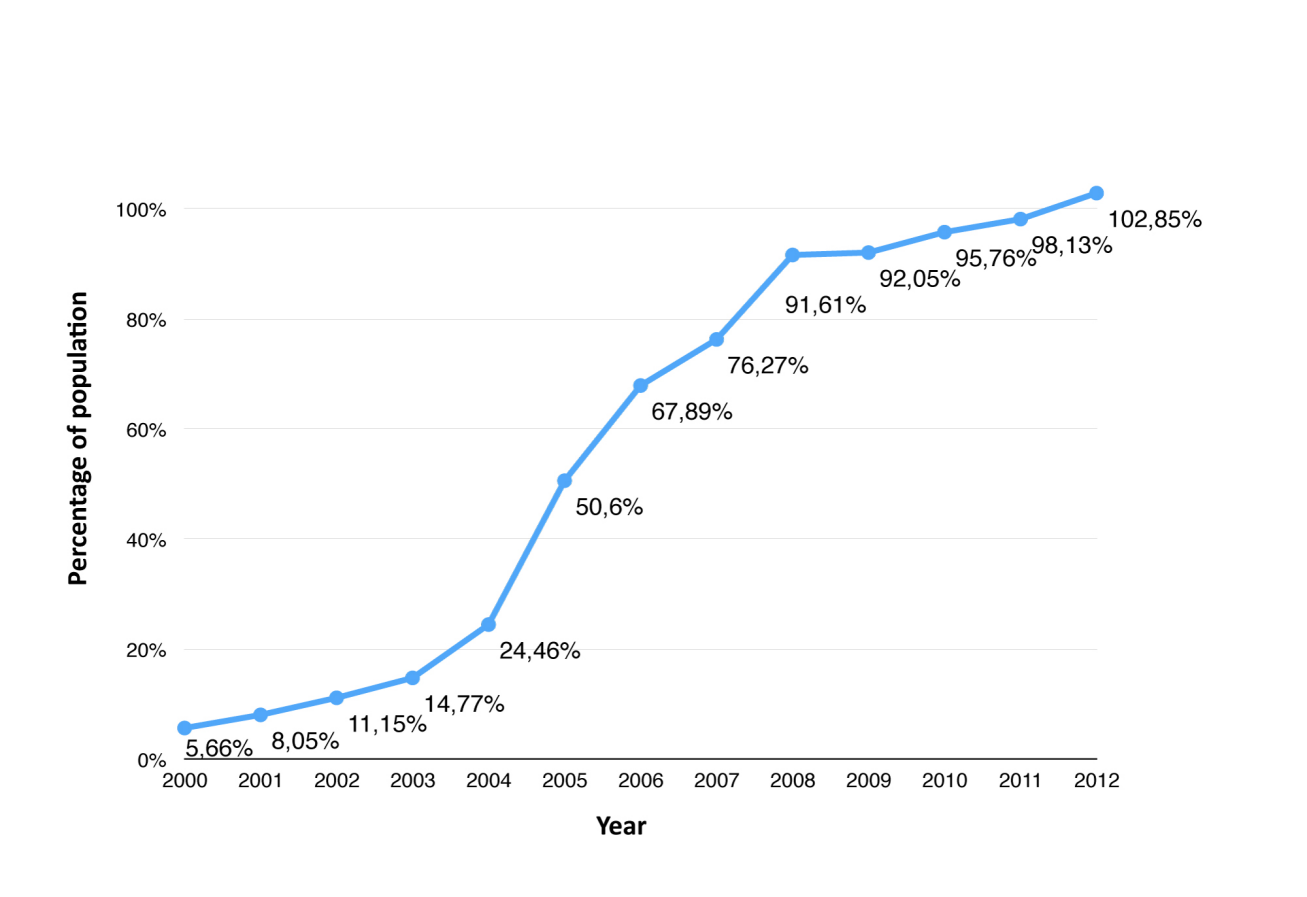
Mobile phone subscriptions in Colombia, 2000-2012. Data taken from the International Telecommunication Union [22].

In Colombia and Latin America in general, penetration of cellular phones among the population reached 100% (Figure 1), and 32% of the population was found to have access to the Internet, including mobile access [22]. Given that the effectiveness of behavioral interventions on sexual health is generally higher with measures on a specific target population and with clear expected outcomes [2], it is of great importance to assess intervention strategies through mobile device apps such as DoctorChat Mobile. Furthermore, medical services in Colombia are costly, and for those under the age of 18, medical appointments can only be scheduled by their parents or their legal guardians. These legal and financial restrictions on the ability of young people to access medical advice are very important when considering the utility of these kinds of strategies, which should be considered to be of high-potential and high-efficacy for the promotion of access to accurate and timely health information among young people.

In this paper we report our experience with the use of DoctorChat Mobile to provide sexual education and information among university students in Bogota, Colombia, and the sexual risk factors detected among them.

## Methods

### Study

**Figure 2:**
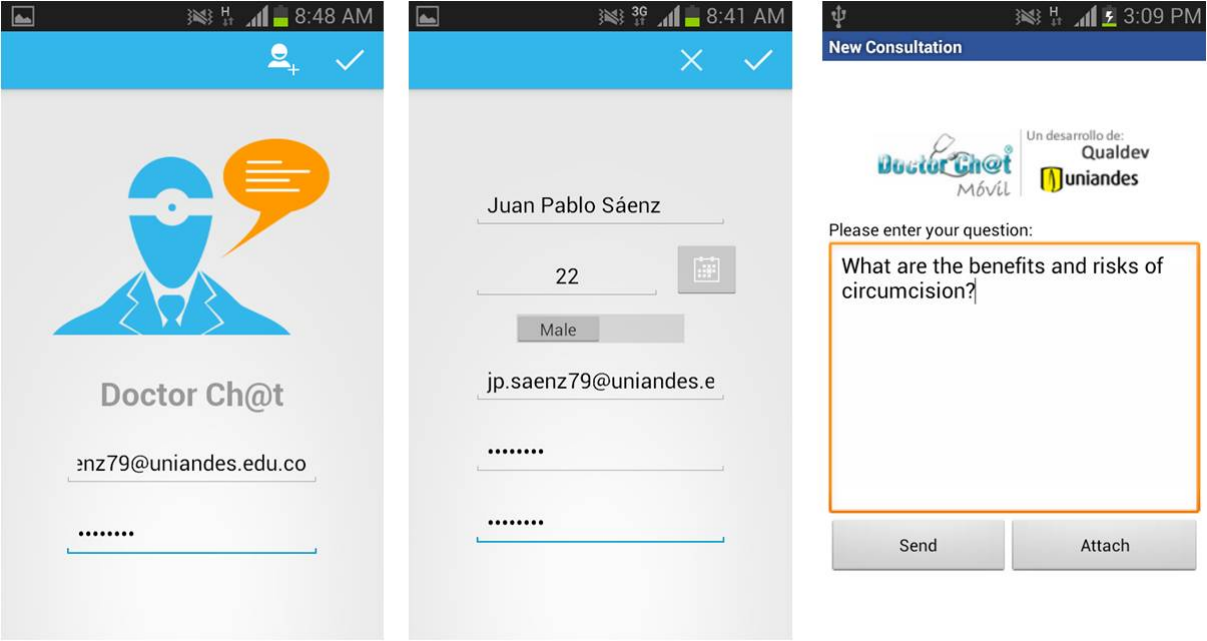
DoctorChat on a mobile device; home screen (left), user profile screen (middle), and free text field (right).

Based on our previous experience with DoctorChat and in alliance with the Systems and Computing Engineering Department of the Universidad de los Andes, we developed an app that allowed users to send inquiries on sexual and reproductive health topics through their mobile phones, and to receive personalized and accurate responses from a knowledgeable group of physicians. Users had to type their questions into a free text field with the possibility of attaching multimedia files (Figure 2). All questions were individually answered asynchronously by the medical team from the Telehealth Center in Fundacion Santa Fe de Bogota with a response rate of 24 to 48 hours. The app ran on 4 mobile platforms (iOS/iPhone, Android, RIM/Blackberry, and Symbian) and 27 mobile devices most used by a cohort of 371 potentially eligible subjects. Potential subjects responded to a short email-based survey which questioned their interest in the project and the type of mobile device they owned.

The reference population was established from our previous experience with the DoctorChat Web platform [19,20]. A nonprobabilistic convenience sampling was performed with the aim of recruiting at least 261 individuals, representing the number of annual average users of DoctorChat posing sexual and reproductive health questions.

### Recruitment and Process

For this study we recruited university students from Universidad de los Andes with access to mobile phones. An invitation to participate was sent via email to a total of 12,463 students from different departments of the university. Participation in the study was voluntary and anonymous. If the subjects decided to participate, they had to register at a specific website, confirm all inclusion and exclusion criteria (Table 1), give their informed consent, and accept the terms and conditions of the project. Once registered, they received an automatic confirmation via email with their personal username and password to access the online survey. After completion of the preintervention online questionnaire [23], they were able to download and use the mobile app.

The recruitment period took 5 months and the app was available for 6 months for each participant. After the 6-month period, participants were invited to complete the postintervention survey, which included questions on satisfaction with the app, to conclude the process.

**Table 1:** Selection criteria for participation in the study.

Inclusion criteria	Exclusion criteria
Aged 18-29 years	Health students and professionals
Reside in Colombia.	People who planned to leave the country for more than 20% of the time available to use the service during the teleconsultation time
Have access to a mobile device that: - allows navigation using a wireless network or an owned data plan, and - allows the installation of the app (installation requirements were provided)	Sex workers.
Have read, understood, and accepted the terms and conditions of the study	Intravenous drug users

### Survey

The survey Family Health International (FHI) Behavioral Surveillance Survey (BSS) for Use With Adults Between 15 and 49 Years [23] was chosen to evaluate knowledge on sexual and other risk behaviors in young adults. This survey has been used and validated for more than 10 years in 20 countries, and has generated significant results in this matter. The BSS was conducted for the first time in Bangkok in 1993, and since then has been conducted mostly on people at high risk of contracting HIV and other sexually transmitted infections in developing countries. The data obtained from these surveys worldwide have enabled the implementation of more robust monitoring and control systems of sexually transmitted diseases such as HIV [23]. The questions were limited to those of the validated survey and were not modified. The second survey included four additional questions on satisfaction with the tool.

### Statistical Analysis

To ensure consistent analysis, data was initially collected and compared with the selection criteria (Table 1). Data with inconsistencies or lacking internal integrity was eliminated. Subsequently, the data was normalized following the second normal form as defined by Kifer et al [24], which ensured consistency and helped with the analysis process by dividing the results into work domains. This normalization also allowed the authors to relate data with the DoctorChat app database, determining which users used the app and how often. Thus, an initial descriptive analysis of the information could be performed.

To perform the inferential statistical analysis, data was divided into three groups based on the nature of the questions on the instrument used. This division permitted comparison of results between the first and second survey. Samples were considered to be independent and, in most cases, followed a normal distribution.

The three analysis groups were as follows: dichotomous data (with positive or negative answers), discrete quantitative data, and ordinal data (such as the Likert scale). For all tests, a confidence interval of 95% was established. For dichotomous-nature data, the Chi-squared test was used, not assuming the Yates correction for continuity. This test was used due to its statistical power and to construct contingency tables. For quantitative data, and given the paired nature of the results, the Student’s *t* test was used, which allows a statistical approach in small samples. This approach allowed the comparison of the variances and means of each sample. Finally, for the ordinal-class data, the measurement scale was established and the Mann-Whitney-Wilcoxon test was used, allowing the determination of differences between two scalar samples given their ranges. All statistical tests were performed using the standard package from the statistical software R (The R Project for Statistical Computing, Institute for Statistics and Mathematics, Wirtschaftsuniversität Wien).

## Results

### Recruitment

**Figure 3:**
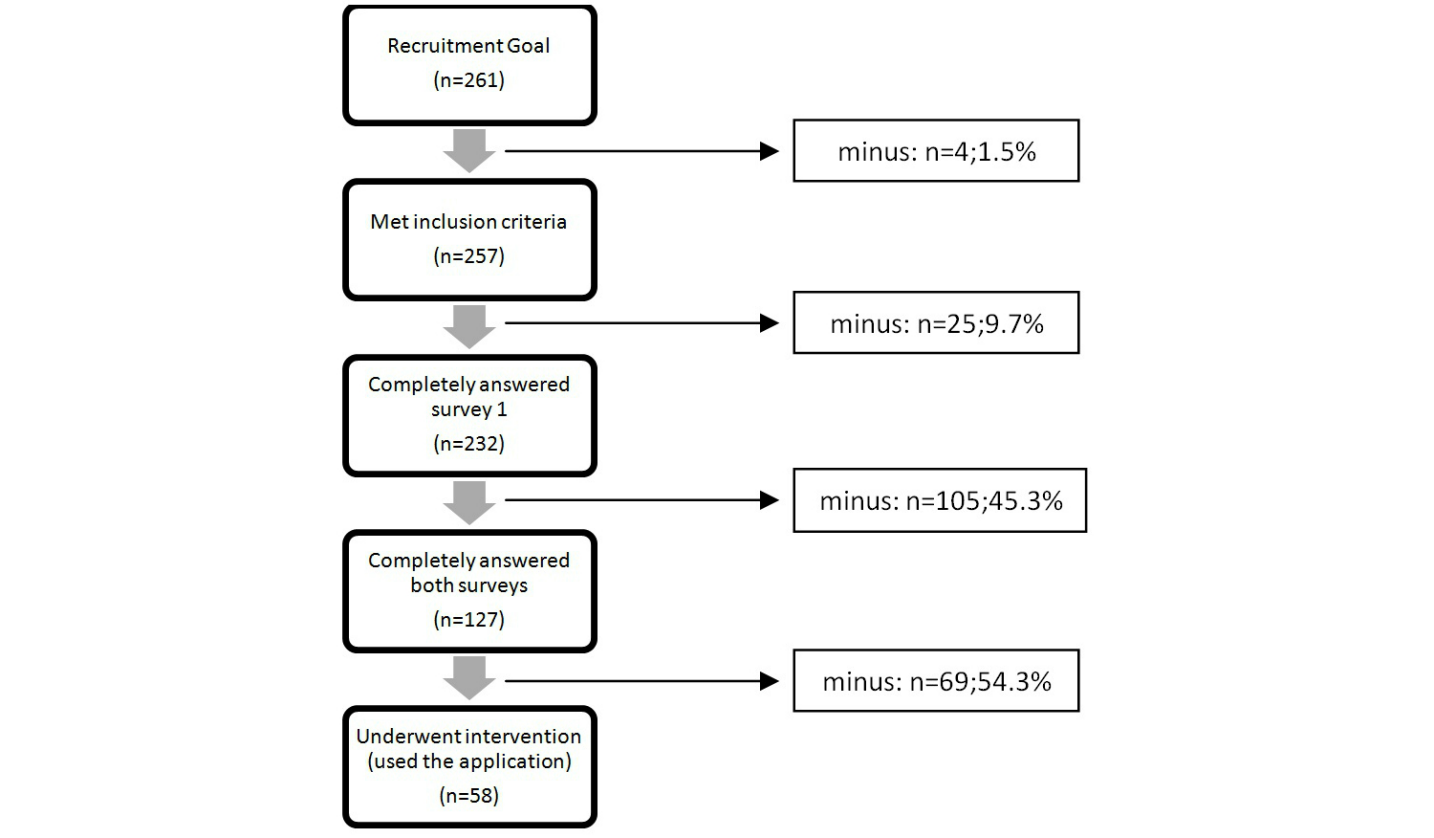
Recruitment and subsets of subjects.

Of the recruitment goal of 261 subjects, 257 registered voluntarily on the virtual platform and met the selection criteria. Of these, 232 completely answered the preintervention survey and, therefore, were eligible to advance to the intervention phase. At the end of the intervention phase, 127 subjects fully answered the postintervention survey. Of the 127 subjects that completed both surveys, 69 of them (54.3%) did not use the mobile app. As the intent was to compare the results from both surveys and their relationship with the use of the mobile tool (intervention), these subjects were excluded, leaving a final effective sample of 58 subjects to be analyzed (Figure 3). As a side analysis, the sexual behavior of those 69 subjects who completed both surveys but did not use the app was performed to compare the results with those who did use the app. In general, there were no statistically significant differences between the groups. Relevant results will be discussed in each subsection.

### Demographic Data

Demographic information was taken from the baseline survey. Of the total analyzable subjects, 53% (31/58) were women, and 47% (27/58) were men. The mean age was 21 years, ranging from 18 to 29 years. The subjects had lived in Bogota (the city where the study was performed) for 13.3 years on average, ranging from 0 to 29 years and most were from mixed ethnic groups (mestizos). Regarding religion, most subjects were Catholic (41/58, 71%), followed by nonreligious affiliation (11/58, 19%). Table 2 summarizes the demographic data from the subjects.

**Table 2:** Demographic information of the subjects (n=58).

		Mean (SD) or n (%)
**Age in years, mean (SD)**		
	Male	20 (2.9)
	Female	21 (4.1)
	Total	21 (3.6)
Years residing in Bogota, mean (SD)		13.3 (9.4)
**Gender, n (%)**		
	Male	27 (47%)
	Female	31 (53%)
	Total	58 (100%)
**Religion, n (%)**		
	Catholic	41 (71%)
	None	11 (19%)
	Don’t know	3 (8%)
	Protestant	2 (3%)
	Other	1 (2%)
	Total	58 (100%)
**Ethnic group, n (%)**		
	Mestizos	41 (71%)
	Caucasian	15 (26%)
	Other	2 (3%)
	Total	58 (100%)

### Consultation Through the Mobile Service

Of the 58 subjects who used the mobile app, 48 of them (83%) consulted 1 to 3 times. Of 58 subjects, 9 of them (16%) made 4 or more consultations, and 1 person (2%) made 29 consultations (Table 3).

**Table 3:** Number of consultations per number of subjects (n=58).

Number of consultations	Number of subjects	%
1	24	41
2	16	28
3	8	14
4	2	3
5	1	2
6	1	2
7	1	2
8	2	3
16	2	3
29	1	2

### Sexual Background

**Figure 4:**
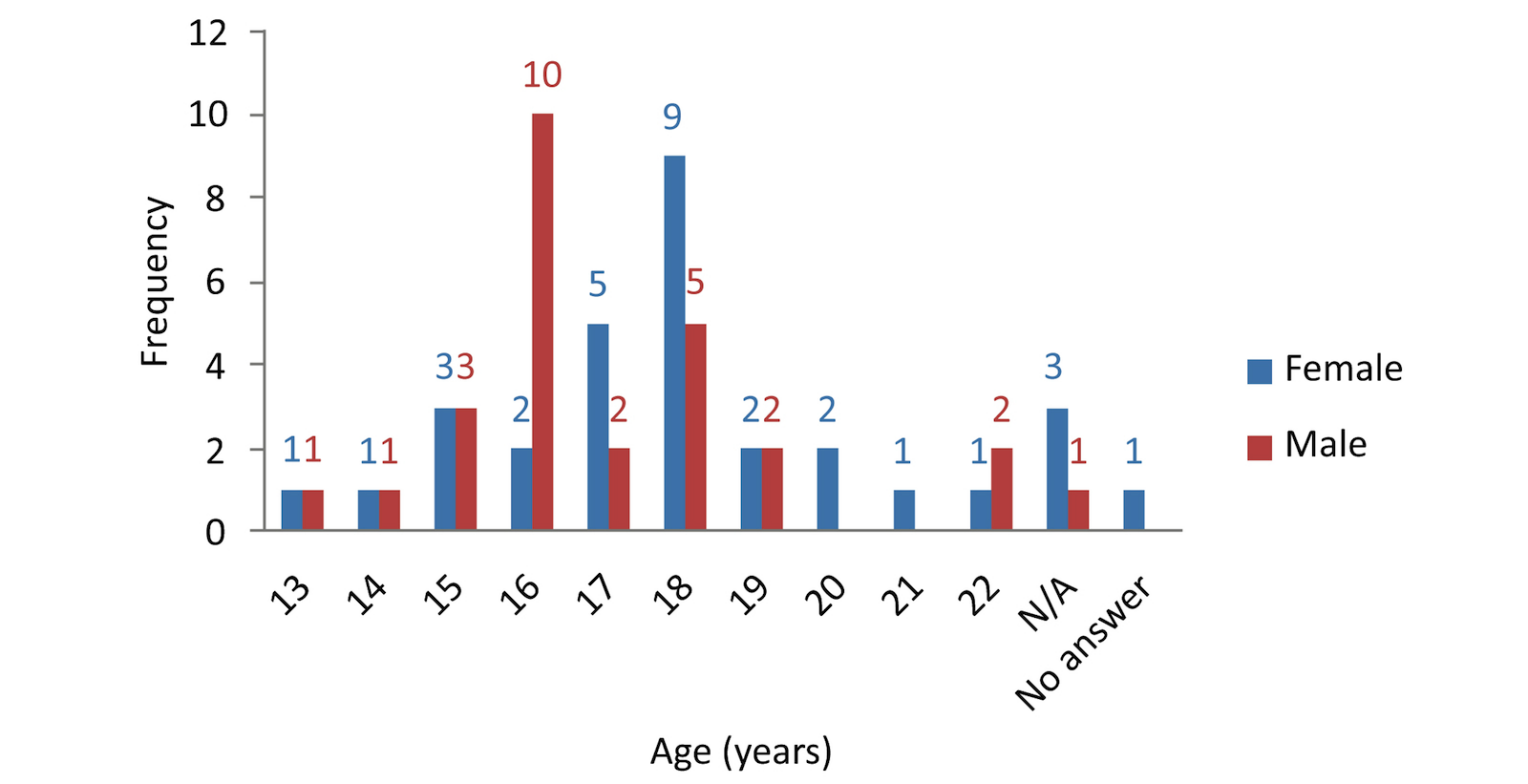
Age and gender comparison of first experience of sexual intercourse.

**Figure 5:**
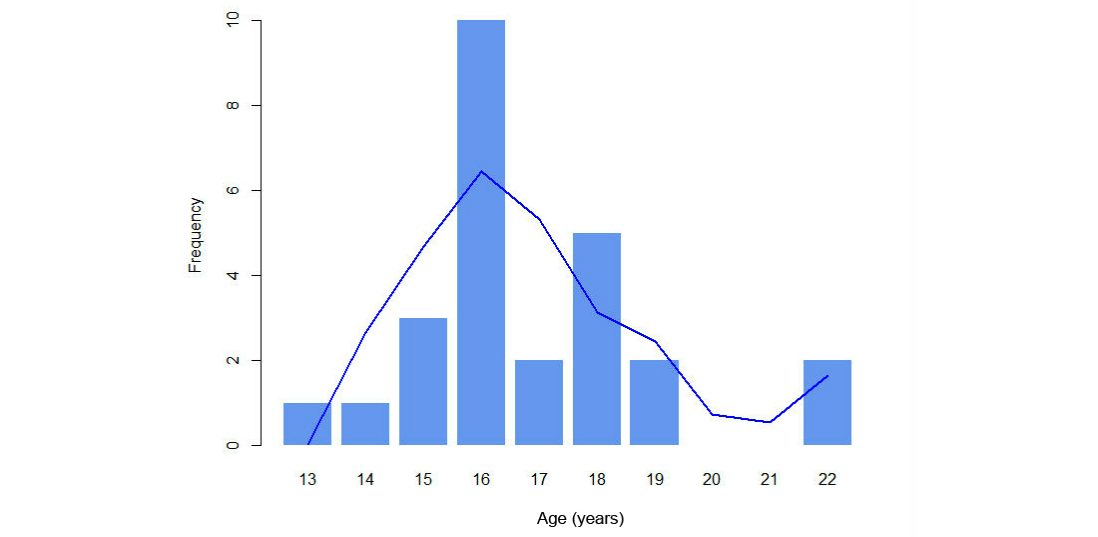
Age of first experience of sexual intercourse for males.

**Figure 6:**
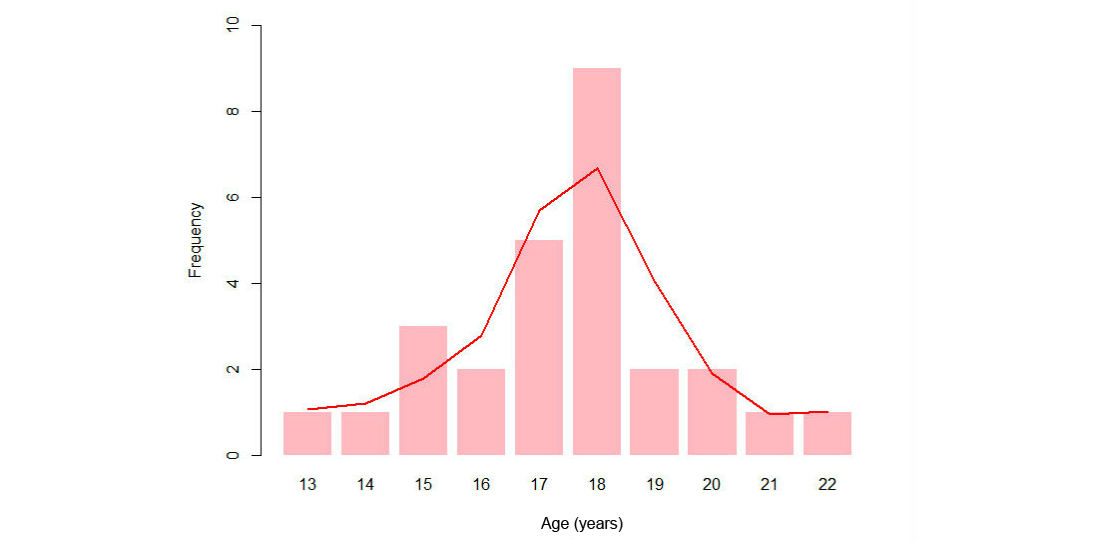
Age of first experience of sexual intercourse for females.

In answer to the question “Have you ever had sex?”, 91% (53/58) replied “yes”, 7% (4/58) replied “no”, and 2% (1/58) did not answer. Of the 58 subjects, 12 women (21%) and 17 men (29%) had sexual intercourse for the first time under the age of 18. Overall, 50% (29/58) of the subjects had sexual intercourse for the first time under the age of 18, ranging between 13 and 22 years, with a mean of 18 years and a standard deviation of 3.3. When comparing both groups, men had sexual intercourse for the first time at a mean age of 16 years, while women started sexual activity at a mean age of 18 years (Figures 4-6).

The differences between those who did not use the app but did complete both surveys (Group 1), and those who did use the app (Group 2) were not statistically significant (*P*=.97), and are shown in Table 4.

**Table 4:** Comparison of age of first sexual intercourse experience between groups.

	Group 1^a^(n=69)	Group 2^b^(n=58)
Answered “yes” to the question “Have you ever had sex?”, n (%)	60 (87)	53 (91)
Age in years of first experience of sexual intercourse, mean (SD)	17(1.9)	18(2.1)
Age range in years^c^	12-23	13-22

^a^Subjects completed both surveys, but did not use the app.

^b^Subjects completed both surveys and used the app.

^c^Age range of first experience of sexual intercourse.

By comparatively analyzing the answers given by the subjects in the preintervention versus the postintervention survey, no statistically significant differences were found. However, risk behaviors among subjects could be identified in both. All results reported in this section are taken from the baseline survey.

Regarding the sexual background of the subjects (Table 5) and the main sexual risk behaviors identified, the surveys demonstrated that over 80% (survey 1, 50/58; survey 2, 47/58) of the subjects had sex in the 6 months prior to the surveys. In the first survey, 2 men out of 27 (7%) claimed to have had sex with another man, whereas in the second survey, 3 out of 27 (11%) did so, with an average of 3 partners in the past 6 months in the second survey. Furthermore, in the first survey, 12 men out of 27 (44%) and 8 women out of 31 (26%) claimed to have had sex with nonregular partners (to whom they were not married, whom they had never lived with, and who did not receive any payment), whereas in the second survey, 13 out of 27 men (48%) and 9 out of 31 women (29%) did so. From these data, there was an average of 2.6 partners for men and 1.6 partners for women. This last difference was statistically significant (*P*=.15). On average, men had sexual intercourse 2.6 times in the past 30 days with their last nonregular partner, and women had sexual relations 2 times (nonstatistically significant difference, *P*=.64 and *P*=.38, respectively). Likewise, 3 men out of 27 (11%) and zero (0%) women claimed to have had commercial sex partners in the last 6 months, with an average of 1.7 commercial partners for men in the last 6 months for the first survey and 3 partners in the second. In the first survey, none of the subjects answered how many times they had sexual intercourse with their last commercial sex partner in the past 30 days. However, in the second survey, the subjects claimed to have had sexual intercourse twice on average with their last commercial partner.

Regarding the use of condoms, 18 subjects out of 58 (31%) reported having sexual intercourse without a condom in the last 6 months. Out of 58 subjects 1 person (2%) reported in the second survey not having used a condom during his last sexual intercourse experience with a commercial partner. Of 58 subjects, 7 (12%) in the first survey and 6 (10%) in the second reported not using a condom the last time they had sexual intercourse with a nonregular partner. Finally, 34% (20/58) of the subjects did not use a condom the last time they had sexual intercourse with their regular partner. Results of sexual background between those who did not use the app and those who did were similar.

**Table 5:** Sexual background of subjects (n=58).

Questions posed in surveys			Survey 1^a^	Survey 2^b^	*P* value
			n or mean (SD)	n or mean (SD)	*P* value
**Have you had sexual intercourse in the last 6 months?, n**					
		Yes	50	47	.23
		No	4	8	
**Commercial Partners** ^c^					
	**Men: Think about the female sexual partners you’ve had in the last 6 months—were any of them commercial?, n**				
		Yes	3	3	N/A^d^
		No	20	20	
	**Both genders: How many commercial partners have you had sex with in the last 6 months?, mean (SD)**				
			1.7 (1.15)	3 (0)	.18
	**Both genders: Think about your most recent commercial sexual partner—how many times did you have sexual intercourse with this person over the last 30 days?, mean (SD)**				
			0 (0)	2 (2.83)	N/A^d^
**Nonregular partners** ^e^					
	**Men: Think about the female sexual partners you’ve had in the last 6 months—were any of them nonregular partners?, n**				
		Yes	12	14	.55
		No	11	19	
	**How many nonregular partners have you had sex with in the last 6 months?, mean (SD)**				
		Men	1.25 (0.46)	1.86 (0.69)	.06
		Women	1.5 (0.84)	1.22 (0.67)	.49
	**Think about your most recent nonregular sexual partner—how many times did you have sexual intercourse with this person over the last 30 days?, mean (SD)**				
		Men	3 (4.44)	2.11 (3.82)	.64
		Women	2.44 (2.24)	1.43 (0.78)	.38
	**Women: Think about the male sexual partners you’ve had in the last 6 months—were any of them nonregular partners?, n**				
		Yes	8	9	.75
		No	17	15	
**Use of Condoms**					
	**Both genders: The last time you had sex with your regular partner, did you and your partner use a condom?, n**				
		Yes	21	21	.91
		No	20	19	
	**Both genders: The last time you had sex with a commercial partner, did you and your partner use a condom?, n**				
		Yes	3	0	.15
		No	2	1	
	**Both genders: The last time you had sex with a nonregular partner, did you and your partner use a condom?, n**				
		Yes	13	15	.66
		No	7	6	
	**Both genders: During the last 6 months, did you ever have sex without a condom with any commercial sexual partner or any other sexual partner with whom you have never lived nor been married to?, n**				
		Yes	18	18	.94
		No	30	29	
**Male homosexual relations** ^f^					
	**Men: Have you ever had any male sexual partners in the last 6 months?, n**				
		Yes	2	3	.81
		No	1	1	
	**Men: How many male partners have you had anal intercourse with in the last 6 months?, mean (SD)**				
			1 (0)	3 (2.64)	.32

^a^Preintervention survey.

^b^Postintervention survey.

^c^Partners with whom you had sex in exchange for money.

^d^Not applicable.

^e^Sexual partners that you are not married to, who you have never lived with, and did not pay—do not include current spouse(s) or live-in sexual partners.

^f^Sexual intercourse defined as penetrative anal sex.

### Sexually Transmitted Diseases

Regarding knowledge of STDs (Table 6), 100% (58/58) of subjects were aware of the existence of STDs. In the first survey, 69% (40/58) of the subjects answered that foul-smelling discharge is a symptom that can occur in both men and women. However, 26% (15/58) answered that this symptom manifests in only men or only women, and 5% (3/58) answered that this symptom does not occur. For this same question in the second survey, 86% (50/58) answered that this symptom can occur in both men and women, 9% (5/58) answered that it manifests in only men or only women, and 5% (3/58) answered that this symptom does not occur. This change represented a statistically significant difference between both surveys (*P*=.04). However, this statistically significant difference was not observed in the group of those who did not use the app (*P*=.25).

Of the 58 subjects, 41% (24/58) in the first survey and 31% (18/58) in the second survey did not consider anal irritation or discharge to be a symptom of STDs.

In the first survey, 19% (11/58) of the subjects answered that itching is a symptom present in only men or only women, and 12% (7/58) answered the same in the second survey.

**Table 6:** Knowledge of sexually transmitted diseases (n=58).

Questions posed in surveys			Survey 1^a^, n	Survey 2^b^, n	*P* value
**Have you ever heard of diseases that can be transmitted through sexual intercourse?**					
	Yes		58	58	N/A^c^
	No		0	0	
**Can you describe any symptoms of STDs in women and/or in men?** ^d^					
	**Genital discharge**				
		Women and men	44	46	.75
		Only in women or only in men	10	8	
		None	4	4	
	**Foul-smelling discharge**				
		Women and men	40	50	.04
		Only in women or only in men	15	5	
		None	3	3	
	**Genital ulcers/sores**				
		Women and men	46	48	.81
		Only in women or only in men	11	5	
		None	1	5	
	**Anal swelling or discharge**				
		Women and men	23	35	.06
		Only in women or only in men	11	5	
		None	24	18	
	**Itching**				
		Women and men	46	47	.99
		Only in women or only in men	11	7	
		None	1	4	
	**Flu-like symptoms**				
		Women and men	25	30	.40
		Only in women or only in men	1	1	
		None	32	27	
	**Abdominal pain**				
		Women and men	11	21	.24
		Only in women or only in men	18	25	
		None	29	12	
	**Burning pain on urination**				
		Women and men	49	48	.74
		Only in women or only in men	8	7	
		None	1	3	
	**Headache**				
		Women and men	20	19	.88
		Only in women or only in men	0	3	
		None	38	36	
	**Diarrhea**				
		Women and men	18	21	.69
		Only in women or only in men	2	1	
		None	38	36	
	**Nausea and vomiting**				
		Women and men	16	19	.93
		Only in women or only in men	10	6	
		None	32	33	

^a^Preintervention survey.

^b^Postintervention survey.

^c^Not applicable.

^d^These questions are meant to assess whether participants think that STD symptoms may occur only in one gender. The questions were taken from the validated FHI survey and were not modified.

### HIV/AIDS

With regard to HIV awareness (Table 7), 1 person out of 58 (2%) claimed not to have ever heard of HIV. Of 58 subjects, 7 individuals (12%) in the first survey and 4 (7%) in the second survey claimed that a person can contract HIV by sharing food with someone who is infected. Out of 58 subjects, 11 (19%) that answered the first survey and 10 (17%) that answered the second survey did not know whether a woman with HIV or AIDS can transmit the virus to her newborn child through breastfeeding.

**Table 7:** HIV knowledge by subjects (n=58).

Questions posed by the surveys		Survey 1^a^, n	Survey 2^b^, n	*P* value
**Have you ever heard about HIV or the disease called AIDS?**				
	Yes	57	58	.32
	No	1	0	
**Can people protect themselves from HIV, the virus that causes AIDS, by using a condom correctly every time they have sex?**				
	Yes	56	57	.32
	No	1	0	
**Can a person get HIV from mosquito bites?**				
	Yes	7	4	.51
	No	42	37	
	Don’t know	8	17	
**Can people protect themselves from HIV by having one uninfected faithful sex partner?**				
	Yes	46	45	.64
	No	8	10	
	Don’t know	0	0	
**Can people protect themselves from HIV by abstaining from sexual intercourse?**				
	Yes	38	41	.64
	No	19	17	
	Don’t know	0	0	
**Can a person get HIV by sharing a meal with someone who is infected?**				
	Yes	7	4	.36
	No	46	48	
	Don’t know	4	6	
**Can a person get HIV by getting injections with a needle that was already used by someone else?**				
	Yes	56	58	.31
	No	1	0	
	Don’t know	0	0	
**Do you think that a healthy-looking person can be infected with HIV, the virus that causes AIDS?**				
	Yes	56	58	.31
	No	1	0	
	Don’t know	0	0	
**Can a pregnant woman who is infected with HIV or AIDS transmit the virus to her baby?**				
	Yes	56	56	.32
	No	0	1	
	Don’t know	1	1	
**Can a woman with HIV or AIDS transmit the virus to her newborn child through breastfeeding?**				
	Yes	20	25	.55
	No	11	10	
	Don’t know	26	23	

^a^Preintervention survey.

^b^Postintervention survey.

### Substance Use

Regarding the use of substances (Table 8), 26% (15/58) of subjects that answered the first survey and 31% (18/58) that answered the second reported consuming alcohol at least once a week in the last 6 months.

Of 58 subjects, 24 (41%) in the first survey and 22 (38%) in the second survey claimed to have used a psychoactive substance at least once. The most common substances used were marijuana (22/58, 38%), tobacco (19/58, 33%), and cocaine (5/58, 9%).

**Table 8:** Substance use by subjects (n=58).

Questions posed by the surveys			Survey 1^a^, n	Survey 2^b^, n	*P* value
**During the last 6 months, how often have you ingested drinks containing alcohol?**					
	Less than once a week		40	36	.49
	At least once a week		15	18	
**Have you ever tried any psychoactive substance?**					
	Yes		24	22	.70
	No		34	36	
**Which of the following, if any, have you tried?**					
	**LSD**				
		Yes	2	5	.17
		No	22	17	
	**Ecstasy**				
		Yes	0	0	N/A^c^
		No	24	22	
	**Cocaine**				
		Yes	5	6	.61
		No	19	16	
	**Marijuana**				
		Yes	23	21	.95
		No	1	1	
	**Tobacco**				
		Yes	19	19	.52
		No	5	3	
	**Poppers**				
		Yes	4	5	.60
		No	20	17	
	**Injectable drugs**				
		Yes	0	0	N/A
		No	24	22	

^a^Preintervention survey.

^b^Postintervention survey.

^c^Not applicable.

### Satisfaction With the Mobile Tool

Of the participants who answered the postintervention survey, 142 of them answered all four satisfaction questions included in the survey. These results are summarized in Table 9. Of these subjects, 92.3% (131/142) of them considered having access to a mobile-based teleconsultation tool on sexual health to be important or very important. Of the respondents, 69.7% (99/142) of them considered the use of the app to be easy or very easy, in contrast with the remaining 30.3% (43/142) that found the app difficult or not so easy to use.

Regarding the subjects’ preferences on learning methods for sexual and reproductive and health topics (degree of perceived effectiveness with the different learning methods), 74.6% (106/142) of the subjects considered that in-person lessons/workshops in a classroom are ineffective and 50.7% (72/142) considered virtual lessons/workshops to be ineffective as well. Of the participants, 54.9% (78/142) thought that a mobile-based teleconsultation tool could be effective, and 54.2% (77/142) had the same perception regarding face-to-face medical consultations. The participants’ overall experiences with DoctorChat Mobile were reported as Excellent, 26.8% (38/142), Good, 50.0% (71/142), Fair, 15.5% (22/142), and Bad, 7.7% (11/142).

Finally, participants were allowed to write free comments about the app. The positive comments alluded to the clarity of the responses, the response time, and the option to send queries anonymously. On the other hand, the negative comments were related to the installation process and the correct use of the tool.

**Table 9:** Satisfaction survey results (n=142).

Questions posed by the satisfaction survey			n	%
**How important is it to you to have access to a mobile teleconsultation tool such as DoctorChat Mobile?**				
	Very important		72	50.7
	Important		59	41.5
	Not important		11	7.7
**How easy was it for you to send queries through your cell phone?**				
	Very easy		43	30.3
	Easy		56	39.4
	Not very easy		32	22.5
	Difficult		11	7.7
**For each of the following four methods, indicate whether you consider it effective or not as a learning method on reproductive and sexual health related topics:**				
	**1. In-person lessons/workshops in a classroom**			
		Effective	36	25.4
		Not effective	106	74.6
	**2. Virtual lessons/workshops**			
		Effective	70	49.3
		Not effective	72	50.7
	**3. Teleconsultation using a mobile device**			
		Effective	78	54.9
		Not effective	64	45.1
	**4. Face-to-face consultation with a health care professional**			
		Effective	77	54.2
		Not effective	65	45.8
**How would you qualify your overall experience with the use of DoctorChat Mobile?**				
	Excellent		38	26.8
	Good		71	50.0
	Fair		22	15.5
	Bad		11	7.7

## Discussion

### Principal Findings

This study provides results regarding not only the use of a mobile teleconsultation service to provide sexual education and information for young adults, but also about sexual risk behaviors among university students in Colombia.

In terms of usage of the app, with a loss rate of 77.8% (58 subjects to be analyzed out of 261) of the subjects and with 41% (24/58) of subjects using the app only once, we can predict, as we have reported in our previous studies, that this behavior reflects the law of attrition described by Eysenbach in 2005 [25]. This law states that eHealth initiatives suffer from a common problem that involves a loss of users over time. This effect is most likely due to a decrease in motivation of the user after their curiosity toward the app has been satisfied by using the app a few times.

When performing the consolidated comparative analysis of changes in sexual risk behaviors pre- and postintervention, most of the results were not statistically significant. However, this study did not intend to change risk behaviors but rather to obtain a descriptive dataset regarding risk behaviors. On this matter, this study confirms that risk behaviors including heterosexual and homosexual unprotected sex, sex with nonregular and commercial partners, substance consumption, and lack of knowledge regarding sexuality are still very frequent problems among young adults regardless of their socioeconomic status [6].

On the other hand, analyzing the details of the effect of the use of the mobile app on sexual behaviors, we noted some interesting issues worth mentioning. It is clear that the use of the tool did not influence sexual practice. Of the 50 subjects who claimed to have had sexual intercourse 6 months prior to the preintervention survey, 92% (46/50) maintained their sexual activity during the postintervention period. Only men reported having had sexual intercourse with commercial partners. It is notable that these men did not answer how many times in the past 30 days they had had sexual intercourse with their ​​last commercial partner in the first survey. However, in the second survey, these men answered this question. Additionally, it is clear that men in this study had more nonregular partners than did women, and this difference was statistically significant.

Likewise, there were no differences regarding sexual practices among homosexual men or the use of condoms. However, there was a difference of only 1 additional person who used a condom with his regular partner between the pre- and postintervention surveys. Moreover, 3 additional subjects reported using condoms with irregular partners in the second survey, and 2 additional subjects reported not using a condom with a commercial partner in the second survey. There were no differences regarding the use of condoms in heterosexual men, with a difference of only 1 additional person who reported having used a condom in the past 6 months with a noncommercial or nonregular partner.

Interestingly, although all subjects had heard about sexually transmitted diseases, 1 person had never heard about HIV/AIDS before the intervention, but this status changed after the use of DoctorChat Mobile. Likewise, 1 person learned that this infection can be prevented with the proper use of condoms. Furthermore, 2 to 3 people learned that HIV/AIDS can be prevented with sexual abstinence, whereas another 3 people learned that the risk of infection is minimal after sharing food with an infected person. In addition, 2 subjects learned that they can become infected after sharing a needle with someone who is infected and at the end of the intervention all participants knew this fact. Similarly, 2 subjects learned that an HIV-infected person can appear healthy, and all subjects were aware of this fact by the end of the intervention.

Paradoxically, 2 additional subjects, after the intervention, did not think they could protect themselves from HIV by having 1 uninfected faithful sex partner, whereas previously they thought they could. Additionally, doubt about the vertical transmission of HIV persisted in the only person who reported doubt at the beginning of the study. However, doubt did not persist in the case of breastfeeding, as 1 to 3 people learned of the causal relationship.

Although the differences in knowledge and risk behaviors for HIV/AIDS were subtle, they are nonetheless important, considering that in 2011 approximately 2.5 million people became infected and 1.7 million died from AIDS-related causes [26]. Other indicators, such as those concerning knowledge of the symptoms of sexually transmitted infections, seemed to improve with the intervention. Specifically, 10 people learned, with the intervention, that malodorous discharge may be a symptom of sexually transmitted infections, which represented a statistically significant difference.

### Comparison With Prior Work

More than 5 years ago, we reported the first experience since the release of DoctorChat, a free-access service of virtual medical orientation in Spanish [19,20]. From September 2006 to March 2007, 270 teleconsultations were received, mostly from women (167/270, 61.9%) and users 18-29 years old (146/270, 54.1%). The main topics of consultation were those related to sexual and reproductive health [19]. Subsequently, the 2-year follow-up of the experience was reported [20]. We observed a tendency similar to that of the first report—between 2007 and 2009, 1624 consultations were received from users, mainly those 18-29 years old (861/1624, 53.02%). The main topic of consultation remained sexual and reproductive health (422/1624, 25.99%). We concluded, in both reports, that the service could be an innovative way to improve community access to health information, particularly sexual and reproductive health. Observing the rapid increase in the spread of mobile devices in Colombia and Latin America, we also concluded that mobile-based interventions could positively impact the delivery of health information. This encouraged us to develop DoctorChat Mobile, an app to support the service of DoctorChat on mobile devices.

Studies like the one conducted by Formigos have shown that "1 in 6 patients consult the Internet before going to the doctor, and 1 in 4 do so afterwards to contrast or complete the information" [27]. On the other hand, the Health Information National Trends Survey (HINTS) [28], a US National Institutes of Health initiative, reported that the first source consulted by patients when consulting specific information is the Internet, despite the fact that the physician is the most trustworthy source of health information.

According to Kirby et al [29], educational strategies aimed at sexual health have a positive impact on sexual risk behaviors without negative effects. This positive impact is associated with increased use of condoms and oral contraceptives, delay in the onset of sexual life, and reduction in the frequency of sexual activity. There are several reasons why young people would request guidance in sexual and reproductive health through a mobile teleconsultation service. The first could be age. Although the computer culture is not yet fully consolidated in Colombia, it is likely that young people make the most use of the Internet to address their health information needs compared with other age groups. Second, in Colombia the parents or legal guardians are responsible for scheduling medical appointments for minors (defined as those less than 18 years of age). Hence, social embarrassment and other limitations may restrict teens from asking their parents or guardians or requesting a medical appointment to solve issues considered taboo, typically those related to sexual and reproductive health. Moreover, and related to the above statement, monetary issues could also be an important factor—few teenagers can afford a private face-to-face consultation. For the group over 18 years old with purchasing power and access to the health care system (which represents the minority), waiting times for a face-to-face consultation can be extensive, and the administrative process necessary to request an appointment can be rather complex. Finally, another possible explanation could be supported by the desire of the users to evaluate the severity of their symptoms before scheduling a face-to-face consultation.

In this context, we think that it is worth insisting on the modeling of strategies for sexual education and guidance aimed at young people through their most-used tools, including mobile devices. As information technologies ultimately become more accessible to the least favored, these strategies could enhance patient empowerment, improve macroeconomic indicators, and achieve Millennium Development Goals such as improving maternal health and combatting diseases such as HIV/AIDS [30].

### Limitations

Regarding the recruitment process, the goal of reaching 261 individuals was not met and the analysis was limited to 58 people, thereby limiting the statistical power to detect changes between both surveys. However, being a descriptive report, the information can be of great interest as a baseline for more robust studies. The demographic characterization of the sample population in this study, in particular the high socioeconomic profile of the participants, can be considered as a limitation. However, as reinforced by the results of this study and other Colombian reports, this population still has sexual risk behaviors and is susceptible to receive innovative sexual educational strategies [3,6].

As for the survey, the original questionnaire was intended for in-person interviews. When applying it online as a self-evaluation tool without an interviewer, some questions could lose their effectiveness. This is particularly evident in those that refer to STD symptoms. The original survey asks openly if the individual can describe any symptoms of STDs in women or men without giving them any evident options. In this study we did not assess whether participants knew that a person could be infected without having symptoms.

Among other limitations of this study, we must mention that the DoctorChat Mobile app works only on smartphones, versus mobile phones with more basic features. This limitation may have contributed to the low use of the mobile teleconsultation service, given that it was necessary to exclude a great percentage of the subjects initially recruited to the study that didn’t meet the technical criteria. Moreover, more than 80% of the 58 subjects that did use the service accessed the app 3 times, and less than 20% of them accessed the service 4 or more times. Unfortunately, the paradoxical situation of telemedicine suggests that the population that is most likely to benefit from such information technology services is the population that has the least access to them [31]. However, this study proves that it is still important to encourage the use of these new technologies among people that can have access to them, given that they are rising steeply in developing countries [14, 22], and given that youngsters are very fond of them. In addition, it’s very important to encourage and spread the use of simpler technologies that have proven to be in some way effective, such as texting or SMS, or in-person educational strategies [11-13].

Thus, it would be useful to repeat this study in other populations that are more vulnerable in terms of age, educational level, and socioeconomic status compared to this study´s subjects. However, this task would involve a mobilization of resources and a much greater budget than that available for our study. Repeating this study in a new population could reveal interesting results, especially considering that in Latin America, at least 30% of women aged 15 to 19 have had some type of sexual experience. Additionally, in Colombia 33% of women under the age of 18 and 70% under 20 have had a sexual experience, with only 7% having used a contraceptive method [32].

Taking into account that obtaining an answer from the DoctorChat Mobile service could take up to 48 hours, it may be inferred that people may not be willing to wait that long for basic information on sexual and reproductive health. However, having access to a professional medical staff giving personalized answers to specific anonymous questions through mobile devices was very well rated among the participants. In addition, most of the positive comments on the app were related to the response time.

### Ethics

This study was conducted with previous approval from Fundacion Santa Fe de Bogota and Universidad de los Andes’ Ethics Committees. In addition, all subjects were provided with an informed consent document and a document containing all terms and conditions of the service prior to their enrollment. This consent was recorded online as the first step in the registration process.

### Conclusions

DoctorChat Mobile seems to be an innovative and well-accepted tool to provide personalized sexual health-related information and education. This study revealed that sexual risk behaviors are frequent among young Colombian adults of high socioeconomic status. This finding confirms the importance of promoting education strategies on this topic and the importance of encouraging the empowerment of young patients with easy access to reliable information, regardless of their origin. However, it would be worthwhile to repeat this study in a more vulnerable population than the one hereby included, such as teenagers with low education levels and socioeconomic status.

DoctorChat Mobile did not result in significant changes in sexual and reproductive risk behaviors in the population studied. Although the recruitment was satisfactory, the loss of subjects was high, and the rate of the use of the service was low. Nonetheless, user satisfaction with the tool was very encouraging and confirmed that these kinds of strategies are well-accepted among young adults, and can be considered as innovative and effective tools to provide accurate and useful health-related information to a new generation of well-informed and empowered patients.

## Abbreviations

AFHS: Adolescent Friendly Health Services
BSS: Behavioral Surveillance Survey
ENDS: National Demographic and Health Survey
FHI: Family Health International
HINTS: Health Information National Trends Survey
SES: socioeconomic status
SMS: short message service
STDs: sexually transmitted diseases
UNFPA: United Nations Fund for Population Activities
WHO: World Health Organization
